# The New National Quarantine Law

**Published:** 1893-04

**Authors:** 


BUFFALO MEDICAL AND SURGICAL JOURNAL
A MONTHLY REVIEW OF MEDICINE AND SURGERY.

*EDITORS:*
THOMAS LOTHROP, M. D. - - WM. WARREN POTTER, M. D.
All communications, whether of a literary or business character, should be addressed to the managing editor: 284 FRANKLIN STREET, BUFFALO, N. Y.

VOL. XXXII. APRIL, 1893. No. 9.

### THE NEW NATIONAL QUARANTINE LAW.

After much debate and delay in the lower house of Congress, the calendar bill No. 1264, entitled, An Act Granting Additional Quarantine Powers and Imposing Additional Duties Upon the Marine Hospital Service, finally passed both houses and received the sanction of the President. It is now, therefore, a law, and succeeds the old National Board of Health law which was passed in 1879. The importance of this matter, and, in view of the probability of the need of this measure during the coming months, warrants a careful review of the provisions of the bill.

The law is divided into nine sections.

Section one makes it unlawful for any ship to enter any port of the United States, except in accordance with the provisions of this act.

Section two requires every vessel clearing for any port in the United States to obtain from the Consular Service of the United States, at port of departure, a bill of health setting forth the sanitary history and conditions of such vessel, and that it has complied with the rules and regulations prescribed for securing the best sanitary condition of said vessel, its cargo, passengers, and crew. The penalty imposed for non-compliance of this provision is not more than $5,000.

Section three directs the Supervising Surgeon-General of the Marine Hospital Service to examine the quarantine regulations of all State and municipal boards of health and to coöperate with them in enforcing such regulations. Where such regulations do not exist, or where additional rules are necessary to prevent the introduction of infectious diseases into the United States, or from one State into another, the Secretary of the Treasury is empowered to draft such regulations, which are to operate uniformly, and in no manner to discriminate against any port or place.

Section four confers upon the Surgeon-General of the Marine Hospital Service, under the direction of the Secretary of the Treasury, the performance of all duties in respect to quarantine and quarantine regulations, to obtain information regarding the spread of infectious diseases, and to publish and transmit to collectors of customs, health officers, etc., weekly abstracts of the consular sanitary reports.

Section five provides that all ships bound for the United States shall take such steps as will place the crew and cargo in the best sanitary condition possible before a bill of health shall be granted them; and, further, that in case of an outbreak of cholera or other contagious or infectious diseases, such ship shall be detained at quarantine for disinfection and isolation.

Section six provides that in case an infected vessel enters a port not provided with the proper facilities for treatment of the same, said vessel must seek the nearest national quarantine station where the necessary accommodations and appliances are provided for disinfection and treatment.

Section seven gives the President power to prohibit the introduction of persons and cargo coming from an infected port whenever there is serious danger of the introduction of cholera or other infectious disease into the United States.

Section eight authorizes the payment of a reasonable sum to the State authorities for all buildings and apparatus surrendered to the United States for quarantine purposes.

Section nine repeals the old National Board of Health law and directs all property belonging to this board to be placed under the control of the Secretary of the Treasury.

The wisdom of passing this bill cannot be questioned and a pity it is that such a law has been so long delayed. The experience of the New York Health Department during the past year demonstrated most vividly the absolute need of a national quarantine law, and, no doubt, had much to do in hastening the enactment of such a measure. Although the Secretary of the Treasury is nominally the absolute head, the Surgeon-General of the Marine Hospital Service is the virtual head of this important measure and so confers upon this department the responsibility which it alone is able to master.

One of the most important provisions is that giving the President power to suspend emigration when the occasion requires. It is to be hoped that this necessity will not arise—especially during the coming summer months.

On the whole, the measure gives confidence and assurance that a vigilant watch will be kept to prevent the entrance into the United States of infectious and contagious diseases.

